# Multiple Resistance Mechanisms to Pyrethroids Insecticides in *Anopheles gambiae sensu lato* Population From Mali, West Africa

**DOI:** 10.1093/infdis/jiaa190

**Published:** 2021-04-27

**Authors:** Moussa Keïta, Nafomon Sogoba, Fousseyni Kané, Boissé Traoré, Francis Zeukeng, Boubacar Coulibaly, Ambiélè Bernard Sodio, Sekou Fantamady Traoré, Rousseau Djouaka, Seydou Doumbia

**Affiliations:** 1 Malaria Research and Training Center, International Center for Excellence in Research, Faculty of Medicine and Odonto Stomatology, University of Sciences, Techniques and Technologies of Bamako, Bamako, Mali; 2 The AgroEcohealth Platform, International Institute of Tropical Agriculture (IITA-Benin), Cotonou, Benin; 3 Faculty of Science and Technique, University of Sciences, Techniques and Technologies of Bamako, Bamako, Mali

**Keywords:** *A gambiae s.l*, insecticide resistance, Mali, pyrethroids

## Abstract

**Background:**

Insecticide-based vector control is responsible for reducing malaria mortality and morbidity. Its success depends on a better knowledge of the vector, its distribution, and resistance status to the insecticides used. In this paper, we assessed *Anopheles gambiae sensu lato* (*A gambiae s.l.*) population resistance to pyrethroids in different ecological settings.

**Methods:**

The World Health Organization standard bioassay test was used to assess F_0_*A gambiae s.l*. susceptibility to pyrethroids. Biochemical Synergist assays were conducted with piperonyl butoxide (PBO), *S,S,S*-tributyl phosphotritioate, and diethyl maleate. L1014F, L1014S, and N1575Y knockdown resistance (kdr) mutations were investigated using TaqMan genotyping.

**Results:**

*Anopheles gambiae sensu lato* was composed of *Anopheles arabienisis*, *Anopheles coluzzii*, and *A gambiae* in all study sites. *Anopheles gambiae sensu lato* showed a strong phenotypic resistance to deltamethrin and permethrin in all sites (13% to 41% mortality). In many sites, pre-exposure to synergists partially improved the mortality rate suggesting the presence of detoxifying enzymes. The 3 kdr (L1014F, L1014S, and N1575Y) mutations were found, with a predominance of L1014F, in all species.

**Conclusions:**

Multiple resistance mechanisms to pyrethroids were observed in *A gambiae s.l*. in Mali. The PBO provided a better partial restoration of susceptibility to pyrethroids, suggesting that the efficacy of long-lasting insecticidal nets may be improved with PBO.

The long-lasting insecticidal nets (LLINs) and indoor residual spraying (IRS) are at the forefront of control strategy against the malaria vector in sub-Sahara Africa. They have been responsible for the progress made in the reduction of malaria burden observed in the past 15 years. However, this progress is being threatened [1] partly because of the resistance of malaria vectors to insecticides. One of the main factors causing this resistance is the wide deployment of the single class of insecticides, the pyrethroids, in both vector control [2] and agriculture for crop protection [3–5]. Indeed, because of their low toxicity for mammalian and rapid insecticidal activity coupled with their repellency or irritant effects, pyrethroids are the first-line recommended insecticides for both health and agricultural sectors [6].

Unfortunately, there are widespread resistance malaria vectors to pyrethroid in many places in Africa [7, 8] and in Mali [9, 10]. Pyrethroid resistance may be the result of either an overproduction of detoxification enzymes (metabolic resistance) [11] or modification of the target site (mutation) [12], reduced insecticide penetration (cuticular resistance) [13], and behavioral change [14]. Pyrethroids and organochlorines have the same site of action and act on the electrical activity of the central and peripheral nervous system of the insects by interacting with the voltage-gated sodium channel (VGSC). This is reflected in the insects by a “knockdown” shock effect [14]. Altered target-site resistance is mediated through knockdown resistance (kdr), involving point mutations in sodium channel genes in the mosquito’s nervous system resulting in cross-resistance to pyrethroids and dichlorodiphenyltrichloroethane [12, 15]. This mutation can lead to the substitution of the leucine with the phenylalanine at the 1014 site (L1014F), or the leucine with the serine (L1014S) [16]. The first one was first described in West Africa and is called *Kdr* West (Kdr-W) [17], and the second was first reported in East Africa [18] and is called Kdr East (Kdr-E). Nowadays, Kdr-W can be found in East [16, 19] and Central Africa [20–22] and Kdr-E can be found in West Africa [23–25]. Besides these 2 mutations, a new one (N1575Y), which appears on the background of Kdr-W and reinforces its action, was reported [26]. This mutation is the substitution of the tyrosine with asparagine at position 1575 of the domain III-IV of VGSC of *A gambiae* [26]. The L1014F and the L1014S mutations have been described in several localities in Mali [10, 27–30], but not the N1575Y mutation.

In the LLNs-based malaria vector control context, close surveillance of vector population susceptibility to pyrethroid and the detection of its underlying mechanisms are essential for insecticides resistance management adapted to the local conditions. In this study, we investigated *Anopheles gambiae sensu lato* (*A gambiae s.l*.) population susceptibility to pyrethroids and the mechanisms underlying phenotypic resistance (target-site mutation and metabolic).

## MATERIALS AND METHODS

### Study Sites

**Figure 1.**
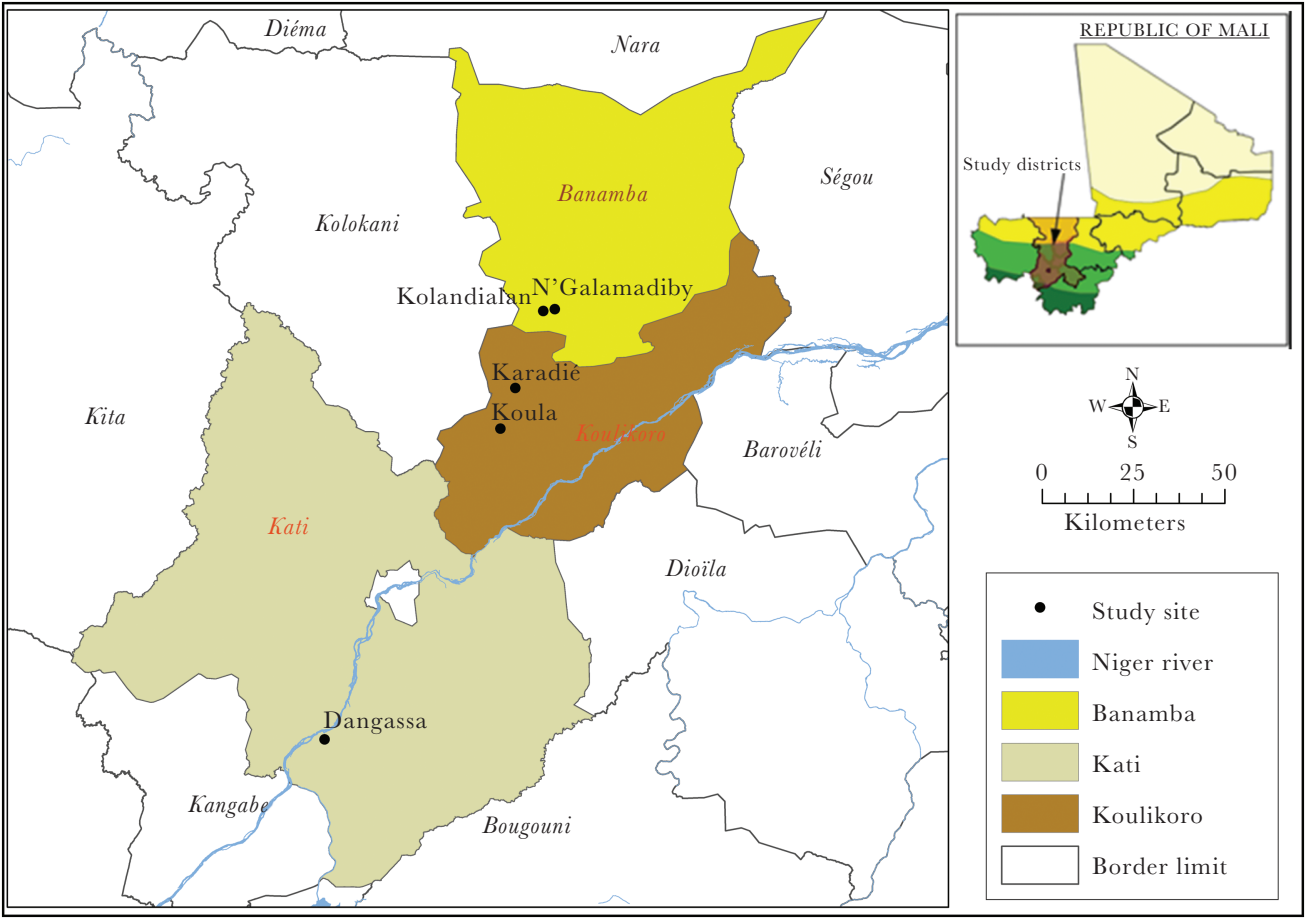
Mali map showing the location of the 5 study sites.

This study was conducted in the following villages: Koula (7.65W, 13.12N) and Karadié (7.60W, 13.24N) in Koulikoro health district; Kolondialan (7.51W, 13.49N) and N’galamadibi (7.48W, 13.48N) in Banamba health district; and Dangassa (8.20W, 12.15N) in the health district of Ouéléssébougou (Figure 1). In each of these localities, herbicides and pesticides are widely used in agriculture. *Anopheles gambiae sensu lato* is the major malaria vector in all of the villages, and vector control strategy is mainly based on the use of LLINs. Since 2014, LLINs coverage was scaled up to universal (2 people for 1 net) coverage through mass-distribution campaigns. From 2008 to 2016, IRS was added to LLINs in Koulikoro, one of the selected health districts of the US President Malaria Initiative in Mali.

### Insecticide Susceptibility Bioassays

*Anopheles gambiae sensu lato* larvae (L1 to L4) and nymphs were collected in various larval breeding sites found in and around each study village by dipping [31] and pooled by site. Collected larvae were then transported and kept in the insectary at the Malaria Research and Training Center (MRTC) in Bamako (temperature, 25–28°C and humidity, 70%–80%) where they were raised to adults. Three- to five-day-old adult female were used for the insecticide susceptibility tests [32]. Insecticides used in this study were deltamethrin (0.05%) and permethrin (0.75%). The number of knockdown mosquitoes was recorded in the course of exposure to the insecticide at 5, 10, 15, 20, 30, 40, 50, and 60 minutes. Survivors after 1-hour exposure were hold in control tubes, kept in the insectary, and fed with 10% sugar solution. Their mortality rate was calculated 24 hours from the end of exposure. Just after exposure, dead specimens were preserved on silica gel, the 24-hour postexposition survivors were preserved in DNALater, and both were kept in a −20°C freezer until deoxyribonucleic acid (DNA) extraction. Synergist tests were performed by exposing mosquitoes to impregnated papers with 4% piperonyl butoxide ([PBO] an inhibitor of oxidases [33]), 0.25% *S,S,S*-tributyl phosphorothioate ([DEF] an inhibitor of esterases), and 8% diethyl maleate ([DEM] an inhibitor of glutathione *S*-transferase [GST]) 1 hour before exposure to deltamethrin and permethrin [32].

### Molecular Identification and Knockdown Resistance Genotyping

Alive and part of the dead mosquito DNAs were individually extracted using the Livak extraction protocol [34]. The species composition of *A gambiae s.l*. (*Anopheles arabiensis*, *Anopheles coluzzii*, and *A gambiae*) was done using the technique of Santolamazza et al [35]. TaqMan SNP genotyping assays for the entire target markers (L1014F, L1014S, and N1575Y) were performed in 10 μL total volume containing 2× quantitative polymerase chain reaction (qPCR) Sensimix (Bioline), 80× primer/probe mix, nuclease-free water, and 1 μL template DNA. Probes were labeled with 2 specific fluorescent dyes, FAM and HEX. The reporter dye, FAM, is used to detect homozygous-resistant genotypes (RR), whereas the quencher fluorescent dye, HEX, is used for the detection of homozygous-susceptible genotypes (SS). Both FAM and HEX are also specific for the detection of heterozygous resistant/susceptible genotypes (RS). Amplifications were performed in an Agilent MX3000 real-time qPCR machine with cycling conditions of 95°C for 10 minutes, followed by 40 cycles at 95°C for 10 seconds and 60°C for 45 seconds. FAM and HEX fluorescence are captured at the end of each cycle, and genotypes are called from endpoint fluorescence using the Agilent MXPro software.

### Statistical Analysis

The bioassays results were calculated as percentage of mortalities with 95% confidence interval of mean and interpreted based on the World Health Organization protocol [32]. Mortalities from synergist-pyrethroid exposure were compared with those obtained from exposure to pyrethroid alone using Pearson χ ^2^ tests, as implemented in GraphPad Prism 8.3.0, with a level of significance set at *P* < .05. The effect of synergists on the mean mortality rates was estimated using the generalized linear mixed model in RStudio 1.2.5033 with a quasibinomial approach. Allelic frequencies of the *Kdr* resistance genes were calculated in dead and alive mosquitoes using the following formula: $\text{F}\left(\text{R}\right)\frac{nRS+2X\left(nRR\right)}{2N}$, where n = total number of mosquitoes carrying a given genotype, RR = total number of homozygote resistant, RS = total number of heterozygote resistant, and N = total number of mosquitoes investigated. A Fisher’s exact test was used in MedCalc easy-to-use online statistical software to test for differences between *Kdr* mutations genotypes in dead and alive mosquitoes [36].

### Ethical Considerations

The protocol of this project has been approved by the Ethics Committee of FMPOS/USTTB under the letter Nº2014/51/CE/FMPOS. The research activities related to this protocol were carried out in accordance with good clinical research practice in humans and good laboratory practice as set out in the international conventions (Helsinki Declaration; International Conference on the Harmonization of Good Practice in Biomedical Research). All of our researchers were trained in good clinical and laboratory practice during the research. In the field, the community (administrative, customary authorities) was informed of all aspects of the study.

## RESULTS

### Distribution of Sibling Species of the *Anopheles gambiae sensu lato* by Site and in Dead and Live Mosquitoes in 2016

A total of 725 specimens of *A gambiae s.l.* derived from sample bioassays including dead and survivors were randomly selected for species identification by PCR. Overall, *A gambiae s.l.* was composed of *A arabiensis* (29.8%), *A coluzzii* (35.9%), and *A gambiae* (34.3%) (Table 1). *Anopheles gambiae* was more frequent in the localities of Karadié, N’Galamadibi, Kolondialan; *A coluzzii* was more frequent in Dangassa; and *A arabiensis* was more frequent in Koula. *Anopheles arabiensis* was the main species in the IRS zone compared with the zone without IRS. The prevalence of *A arabiensis* was significantly higher (χ ^2^ = 13.79, *P* = .0002) in the IRS zone (44.9%, N = 123) compared with the non-IRS zone (20.6%, N = 93%). In contrast, *A coluzzii* was the most prevalent species (χ ^2^ = 9.21, *P* = .0024) in the non-IRS zone (44%, N = 199) compared with the IRS zone (22.3%, N = 61). For *A gambiae*, there no significant difference (χ ^2^ = 0.230, *P* = .6316) between the IRS (32%, N = 90) and the non-IRS zone (35%, N = 159).

**Table 1.** Species Composition of 3 Members of the *Anopheles gambiae sensu lato* in 5 Localities in Mali in 2016

Localities	*Anopheles arabiensis*		*Anopheles coluzzii*		*Anopheles gambiae*	
	N	%	N	%	N	%
Koula	91	49.7	46	25.1	46	25.1
Karadié	32	35.2	15	16.5	44	48.4
Kolondialan	24	26.1	31	33.7	37	40.2
N’Galamadibi	58	32.0	44	24.3	79	43.6
Dangassa	11	6.2	124	69.7	43	24.2
Total	216	29.8	260	35.9	249	34.3

### Resistance Phenotypes and the Effect of Pre-Exposure to Synergists

**Figure 2.**
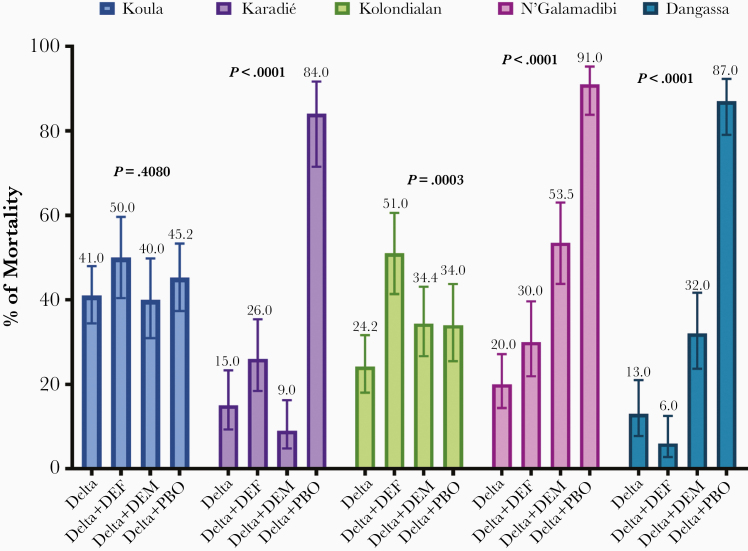
Mortalities recorded in the *Anopheles gambiae sensu lato* populations from Mali in 2016.

**Figure 3.**
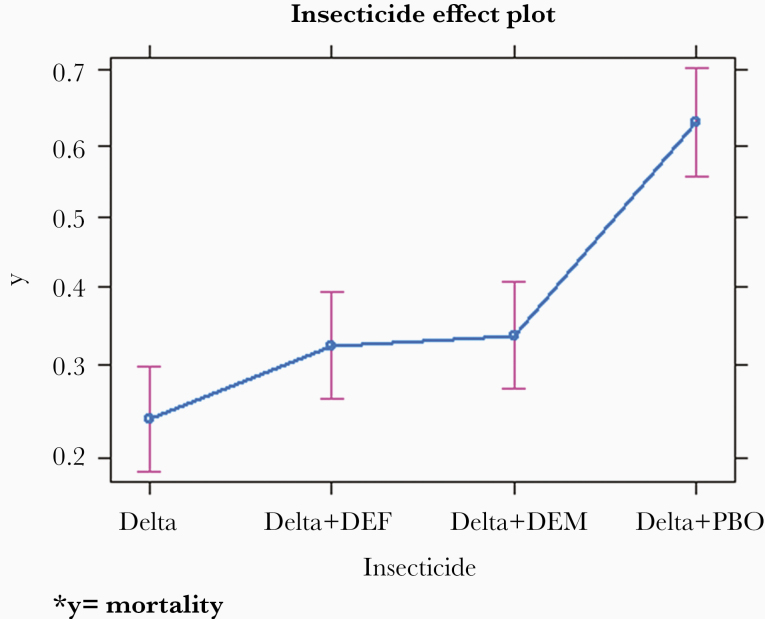
Effect of synergists on the mean mortality of *Anopheles gambiae sensu lato* in the study area. *, y = mortality. Delta, deltamethrin; DEF, *S,S,S*-tributyl phosphorothioate; PBO, piperonyl butoxide.

Before starting with the susceptibility bioassays, we performed a bioefficacy test of the insecticide-impregnated papers using the known susceptible strain of Kisumu, where 100% mortality was observed for both permethrin insecticide-impregnated papers. In the localities where tests were performed, we observed a strong phenotypic resistance to permethrin, with mortality rates ranging from 25% to 43.5%. A total of 26 replicates of susceptibility bioassay tests (8 with deltamethrin alone and 18 with synergist DEF, DEM, or PBO) were performed. In all of the replicate tests, mortality rates in controls did not exceeded 2%. Strong phenotypic resistance was observed in all study sites with deltamethrin alone (Figure 2). There was a significant difference (*P* < .01) when comparing mortality rates with deltamethrin alone and synergist deltamethrin at all sites except for Koula village, where no significant difference was observed (*P* = .4080). The PBO-deltamethrin association showed a significant increase (*P* < .01) in mortality rates at Karadié (84.0% vs 15.0%), N’Galamadibi (91.0% vs 20.0), and Dangassa (87.0% vs 13.0%), suggesting the implication of metabolic resistance (oxydases) in addition to the *Kdr* mutations. The slight increase in mortality (51.0% vs 24.2%) observed at Kolondialan with the DEF deltamethrin suggests the implication of esterase resistance mechanism at this site. In summary, among the different synergists tested, the PBO showed the highest increase in the overall mortality of the *A gambiae s.l*. population (*P ≤* .01) in the 5 study sites (Figure 3).

The limited number of larvae obtained in the field did not allow us to perform susceptibility bioassay tests with permethrin alone and/or synergist permethrin in all localities. In the localities where tests were performed, we observed a strong phenotypic resistance to permethrin and deltamethrin, with mortality rates ranging from 25% to 43.5%.

### Genotyping and Allelic Distribution of Knockdown Resistance L1014F and L1014S Mutation in the *Anopheles gambiae sensu lato* Population From All Sites in 2016

The L1014F mutation was detected in the 3 species of *A gambiae s.l.* (*A arabiensis*, *A coluzzii*, and *A gambiae*). There was site-to-site variation in the resistance allele frequency, with the highest being observed in Dangassa for all species (Table 2). The overall frequency of the resistant allele was significantly higher (*P* < .01) in *A coluzzii* (43.2%) and *A gambiae* (42.6%) compared with *A arabiensis* (16.3%).

**Table 2.** Variation in the Allelic Frequencies of the *Kdr* Mutations in Members of *Anopheles gambiae sensu lato* by Study Sites in Mali in 2016

			*Anopheles arabiensis*				*Anopheles coluzzii*				*Anopheles gambiae*			
			Genotypes			%	Genotypes			%	Genotypes			%
Types	Area	Localities	RR	RS	SS	Freq	RR	RS	SS	Freq	RR	RS	SS	Freq
L1014F	IRS	Koula	3	10	72	9.41	4	24	18	34.78	4	21	21	31.52
		Karadié	0	15	16	24.19	0	8	6	28.57	0	36	6	42.86
	No IRS	Kolondialan	0	14	9	30.43	0	23	7	38.33	0	34	2	47.22
		N’Galamadibi	0	13	36	13.27	5	18	14	37.84	1	51	12	41.41
		Dangassa	0	7	4	31.82	17	92	15	50.81	7	29	5	52.44
		Total	3	59	137	16.3	26	165	60	43.2	12	171	46	42.6
L1014S	IRS	Koula	3	23	60	16.86	0	3	42	3.33	0	5	36	6.10
		Karadié	0	2	29	3.23	0	0	15	0.00	1	1	42	3.41
	No IRS	Kolondialan	0	0	24	0.00	0	0	30	0.00	0	0	37	0.00
		N’Galamadibi	1	8	48	8.77	1	4	38	6.98	0	2	77	1.27
		Dangassa	0	0	10	0.00	0	1	118	0.42	0	0	39	0.00
		Total	4	43	171	11.7	1	8	243	2.0	1	8	231	2.1
N155Y	IRS	Koula	0	1	89	0.003	0	6	40	6.52	0	3	42	3.33
		Karadié	0	3	28	4.83	0	1	14	3.33	0	10	32	11.90
	No IRS	Kolondialan	1	4	15	15.00	0	3	23	5.76	3	11	21	24.28
		N’Galamadibi	0	0	51	0.00	0	9	29	6.54	0	9	60	6.54
		Dangassa	0	0	11	0.00	0	41	83	16.53	0	9	34	9.37
		Total	1	8	194	2.5	0	60	189	12.1	3	42	189	10.3

Abbreviations: Freq, frequency; IRS, indoor residual spraying; RR, homozygous-resistant genotype; RS, heterozygous resistant/susceptible genotype; SS, homozygous-susceptible genotype.

In contrast with its sister mutation L1014F, the frequency of the resistance allele of the L1014S *Kdr* mutation was significantly higher (*P* < .01) in *A arabiensis* (11.7%) compared with *A coluzzii* (2.0%) and *A gambiae* (2.1%). Koula site showed the highest frequency (16.86%) of this allelic frequency, which was not detected in Kolondialan.

The N1575Y *Kdr* mutation was detected in all sites, and in all the species, except for *A arabiensis* in N’Galamadibi and Dangassa. Overall, the resistance allele frequency of this mutation was significantly higher (*P* < .01) in *A coluzzii* (12.1%) and *A gambiae* (10.3%) compared with *A arabiensis* (2.5%).

### Role of L1014F, L1014S, and N1575Y Mutations in Pyrethroid Resistance

We observed a change in the allelic frequency of the 3 Kdr mutations in the dead and the survivors after exposure to the insecticide in all localities and all species (Tables 2–4). The L1014F mutation was present in *A arabiensis* in all localities with a higher allelic frequency in survivors compared with dead mosquitoes in almost all sites except Karadié and N’Galamadibi; however, there was no statistical difference between their allelic frequencies (*P* > .05). Still, in *A arabiensis*, the L1014S mutation was absent in Kolondialan and Dangassa in the dead and survivors and only in the Karadié dead. The L1014S frequency in survivors of A arabiensis was higher than in dead specimens in Koula and N’Galamadibi. However, there was no statistical difference (P > .05). The N1575Y mutation was absent in the *A arabiensis* specimens from the localities of Dangassa and N’Galamadibi. It was present in the other sites with allelic frequencies varying between 1.11% and 15.19% (Table 4). The frequency of the L1014F mutation in *A coluzzii* was higher in postexposure survivors than in the dead (Table 3). However, we did not observe a statistical difference between the 2 proportions (*P* > .05). On the other hand, this mutation was not present among the dead in Karadié. The L1014S mutation was not found in A coluzzii at Karadié and Kolondialan. In N’Galamadibi and Dangassa, it was found only in our samples of *A coluzzii* dead. In Koula, the frequency of L1014S was higher among the dead than among the survivors. N1575Y was present in survivors of *A coluzzii* populations and absent in samples taken after death in this species (Table 4) to Koula, Karadié, and Kolondialan. The frequency of the N1575Y R allele was greater in survivors compared with deaths. However, there was no statistical difference between these 2 frequencies in N’Galamadibi and Dangassa (*P* ≥ .3232).

**Table 3.** L1014F and L1014S Allele Frequencies in Alive and Dead Samples of 3 Species of the *Anopheles gambiae sensu lato* After Exposure to Deltamethrin

Localities	*Anopheles arabiensis*				*Anopheles coluzzii*				*Anopheles gambiae*			
	% Kdr-W Freq (N)		Kdr-E Freq (N)		Kdr-W Freq (N)		Kdr-E Freq (N)		Kdr-W Freq *(N)*		Kdr E Freq (N)	
	Alive	Dead	Alive	Dead	Alive	Dead	Alive	Dead	Alive	Dead	Alive	Dead
Koula	13.4 (41)	5.7 (44)	22.2 (45)	10.9 (41)	42.7 (34)	12.5 (12)	1.5 (34)	9.1 (11)	48.1 (26)	10.0 (20)	4.6 (22)	7.9 (19)
Karadié	23.9 (23)	25.07 (8)	4.4 (23)	0.0 (8)	30.8 (13)	0.0 (1)	0.0 (14)	0.0 (1)	44.9 (39)	16.7 (3)	3.7 (41)	0.0 (3)
Kolondialan	50.0 (1)	29.6 (22)	0.0 (1)	0.0 (23)	47.5 (20)	20.0 (10)	0.0 (20)	0.0 (10)	50.0 (29)	35.7 (7)	0.0 (29)	0.0 (8)
N’Galamadibi	11.1 (27)	15.9 (22)	10.0 (30)	7.4 (27)	40.6 (16)	35.7 (21)	0.0 (20)	13.0 (23)	44.4 (45)	34.2 (19)	0.0 (57)	4.6 (22)
Dangassa	50.0 (2)	27.8 (9)	0.0 (2)	0.0 (8)	52.3 (108)	40.6 (16)	0.0 (103)	3.1 (16)	55.6 (36)	30.0 (5)	0.0 (33)	0.0 (6)

Abbreviations: Freq, frequency; Kdr-E, knockdown resistance East; Kdr-W, knockdown resistance West.

**Table 4.** N1575Y Allele Frequencies in Alive and Dead Samples of 3 Species of the *Anopheles gambiae sensu lato* After Exposure to Deltamethrin

Localities	*Anopheles arabiensis*		*Anopheles coluzzii*		*Anopheles gambiae*	
	N1575Y Freq (N)		N1575Y Freq (N)		N1575Y Freq (N)	
	Alive	Dead	Alive	Dead	Alive	Dead
Koula	0.00 (45)	1.11 (45)	11.76 (34)	0.00 (12)	6.00 (25)	0.00 (20)
Karadié	6.52 (23)	0.00 (8)	3.57 (14)	0.00 (1)	12.50 (40)	0.00 (2)
N’Galamadibi	0.00 (26)	0.00 (25)	17.65 (17)	7.14 (21)	6.00 (50)	7.89 (19)
Kolondialan	0.00 (1)	15.79 (19)	7.89 (19)	0.00 (7)	17.24 (49)	58.33 (6)
Dangassa	0.00 (2)	0.00 (9)	17.13 (108)	12.50 (16)	9.46 (37)	16.67 (6)

Abbreviations: Freq, frequency.

The frequency of the Kdr-W mutation in *A gambiae* (Table 3) was higher in survivors compared with that in all sites except Koula, with no statistical difference observed (*P* > .05). In Koula, it was statistically higher in the surviving populations compared with that of deaths (*P* = .0064). The Kdr-E was missing from our *A gambiae* samples in Kolondialan and Dangassa. In Koula, its allelic frequency was greater among the dead than among the survivors without statistical difference (*P* = .6598). On the other hand, in Karadié, the Kdr-E was absent from the specimens of *A gambiae* dead. The N1575Y mutation was absent from the Koula and Karadié insecticide samples (Table 4). The frequency of its resistant allele was significantly greater in deaths compared with the survivors of *A gambiae* (*P* > .05) in Kolondialan and Dangassa.

## DISCUSSION

In this study, we investigated the susceptibility of *A gambiae s.l.* populations to pyrethroids (permethrin and deltamethrin) and determined the resistance mechanisms (target site mutation and metabolism) underlying the phenotypic resistance in 3 health districts. We observed a high phenotypic resistance of *A gambiae s.l*. in all investigated sites, which is in line with the current trend of pyrethroid resistance spreading in the major malaria vectors across the continent [21, 37–41]. The previously reported explanation for this has been the selection pressure due to the widespread use of pyrethroids in both malaria vectors and agricultural pests control [39, 42–44]. Indeed, the wide deployment of pyrethroid-based control tools in vector control and the noncompliance with best agricultural pesticides management practices by farmers exercise constant selection pressure on anopheline mosquitoes [45].

Among the different synergists tested, the PBO (inhibitor of cytochrome P450 enzymes) showed an important partial restoration of *A gambiae s.l.* population sensitivity to deltamethrin in Karadie, N’Galamadibi, and Dangassa. A slight, partial restoration was also observed with the DEF (inhibitor of esterases) at Kolondialan and DEM (inhibitor of GST) at N’Galamadibi. This suggests the presence of all 3 metabolic resistance mechanisms in 1 or most of our study sites in addition to the different *Kdr* mutations (responsible for pyrethroids insecticide resistance) [33]. Cytochrome P450 is implicated the most among the 3.

The 3 species of *A gambiae s.l*. (*A gambiae*, *A coluzzii*, and *A arabiensis*) were found in sympatry in the different study localities as commonly reported in Mali [10, 46]. However, there was a variation in their frequency distribution by localities because of variation in local conditions, with each species having specific preferences. *Anopheles coluzzii* was the predominant species in Dangassa, and *A gambiae* was the predominant species in Karadié, N’Galamadibi, and Kolondialan. The diversity of the composition of *A gambiae s.l.* in the study sites could be due to an interspecific exclusion competition between the 3 species [47]. The high frequency of A coluzzii in Dangassa could be due to the presence of the flooding plain between the village and the River Niger used for rain-fed rice growing that offer favorable permanent and semi-permanent breeding habitats for this species [41, 46]. Several works in Mali and elsewhere have shown the predominance of *A coluzzii* in rice-growing areas [25, 48–50]. The predominance of *A gambiae* and *A arabiensis* in Karadié, N’Galamadibi, and Kolondialan could be explained by the presence of numerous favorable breeding sites (such as brick pits and puddles) for the development of these species.

Our study showed a high *Kdr_W* (L1014F) resistance allele frequency in all species at all sites. This is consistent with the results observed in several West African countries [30, 40, 51, 52]. The *Kdr_E* (L1014S) resistance allele was also detected in the 3 species of *A gambiae s.l.* This allele, originating from East Africa, was recently reported in many Central and West African countries [23, 25, 53, 54]. Recent studies have reported its presence in *A gambiae* and *A coluzzii* in Mali [29, 55]. However, to our knowledge, this is the first report of its presence in wild *A arabiensis*.

Our data showed a relatively high prevalence of the 1575Y resistance allele in *A coluzzii* and *A gambiae* species, as recently reported by Mavridis et al [55] in Mali and in several other countries of West Africa [24–26, 56]; however, this is the first report in *A arabiensis* specimens in Mali. The presence of the 3 *Kdr* mutations coupled with the partial restoration of susceptibility when mosquitoes were pre-exposed to PBO indicate the involvement of both molecular and metabolic resistance mechanisms to pyrethroids in Mali.

## CONCLUSIONS

This study showed a widespread and high phenotypic resistance of *A gambiae s.l.* species to pyrethroids. Both target-site mutation and metabolic resistance mechanisms were underlying this resistance in the 3 species of *A gambiae s.l.* in Mali. Besides the *Kdr_W* mutation, to our knowledge, this is the first report to describe the presence of the N1575Y and the *Kdr E* in *A arabiensis* in Mali. A process that includes a good insecticide resistance management strategy under a multisectoral system is needed to mitigate the spread of multiple resistance mechanisms of malaria vectors insecticides in Mali.

## Notes

***Acknowledgments*.** We thank the National Malaria Control Programme for support and the population of the study sites for collaboration. We also thank all of the Malaria Research and Training Center staff who supported for the field work.

***Authors contributions*.** M. K. researched the hypotheses, contributed to data collection and analysis, and wrote the manuscript. N. S. researched the hypotheses and corrected and approved the final version of the manuscript before submission. F. K. contributed to data collection and analysis of bioassay data. B. T. contributed to data collection and analysis of bioassay data. F. Z. contributed to molecular analyses. B. C. contributed to data collection. A. B. S. contributed to data collection. R. D. corrected and approved the final version of the manuscript before submission. S. F. T. corrected and approved the final version of the manuscript before submission. S. D. researched the hypotheses and corrected and approved the final version of the manuscript before submission.

***Financial support*.** This work was funded by the Special Programme for Research and Training in Tropical Diseases (TDR) of the World Health Organization under the grant number B20388 with the support from the National Institutes of Health through the West African International Center of Excellence for Malaria Research (ICEMR), National Institute of Allergy and Infectious Diseases (NIAID) grant numbers: U19AI089696, U19AI129387, and D43TW008652.

***Supplement sponsorship***. The supplement is sponsored by TDR, the Special Programme for Research and Training in Tropical Diseases, based at the World Health Organization.

***Potential conflicts of interest*.** All authors: No reported conflicts of interest. All authors have submitted the ICMJE Form for Disclosure of Potential Conflicts of Interest.
